# Proteostasis signatures in human diseases

**DOI:** 10.1371/journal.pcbi.1013155

**Published:** 2025-06-17

**Authors:** Christine M. Lim, Michele Vendruscolo

**Affiliations:** Centre for Misfolding Diseases, Yusuf Hamied Department of Chemistry, University of Cambridge, Cambridge, United Kingdom; Universita degli Studi di Torino, ITALY

## Abstract

The protein homeostasis (proteostasis) network maintains the proteome in a functional state. Although this network has been comprehensively mapped, its perturbations in disease remain incompletely characterised. To address this problem, here we define the proteostasis signatures, which represent the characteristic patterns of change in the proteostasis network associated with disease. We performed a large-scale, pan-disease analysis across 32 human diseases spanning 7 disease types. We first identified unique proteostasis perturbations in specific disease states. We then uncovered distinctive signatures differentiating disease types, pointing to a range of proteostasis mechanisms in disease development. Next, we tracked the temporal evolution of proteostasis signatures, revealing shifts in proteostasis disruption over the course of disease progression. Finally, we demonstrated how smoking, a major risk factor for many diseases, impairs proteostasis in a manner similar to disease, potentially creating a predisposed environment for disease onset. These results illustrate the opportunities offered by the study of human diseases from the perspective of proteostasis signatures.

## Author summary

The maintenance of proteostasis is critical for optimal cellular function and organismal health. Although the dysregulation of the proteostasis network has been implicated in diverse human diseases, the characteristic patterns of this disruption across diseases remain poorly understood. Here, we apply the concept of proteostasis signatures, which describe distinct molecular alterations in the proteostasis network associated with specific diseases. Through a large-scale analysis encompassing 32 diseases across seven categories, we identify unique proteostasis perturbations that differentiate disease types, reveal temporal dynamics in disease progression, and highlight lifestyle factors, such as smoking, that exacerbate proteostasis collapse. These findings provide a unified framework for understanding disease pathogenesis, identifying biomarkers, and developing targeted therapies to restore proteostasis.

## Introduction

The proteostasis network coordinates the synthesis, folding, trafficking, and degradation of proteins [1–3]. This intricate network encompasses molecular chaperones, degradation pathways, and regulatory systems that collectively ensure the proper maintenance of the proteome [1–3]. Maintaining the balance between these processes is essential for optimal cellular function, and its disruption has been implicated in numerous diseases, including cancer, neurodegenerative disorders, and autoimmune conditions [4–7]. As proteins misfold, are damaged and fail to be degraded, cells face increased stress and dysfunction, contributing to disease pathogenesis [4–7]. Understanding the mechanisms underlying proteostasis impairment is critical for developing therapeutic strategies that restore cellular balance [4–7].

Proteostasis dysregulation has been shown to manifest in distinct patterns across different diseases, reflecting the diversity of underlying mechanisms [1–5]. For example, neurodegenerative conditions such as Alzheimer’s and Parkinson’s diseases are characterized by the progressive aggregation of misfolded proteins, whereas cancers exploit proteostasis network like the ubiquitin-proteasome pathway to sustain rapid cell division [1–5] and molecular chaperones, which have been implicated in multiple hallmarks of cancer [8] and correlated with poorer prognosis [9–12]. While these disease-specific patterns are well-recognized, their broader significance as systematic signatures of proteostasis dysfunction has yet to be fully elucidated.

Inspired by the impact of the study of mutational signatures in cancer research [13–15], which have identified the molecular drivers of tumor biology and guided targeted therapies, we investigated a similar approach for understanding proteostasis disruption across diseases. Proteostasis pathways are known to be intricately linked to many diseases, each characterized by unique patterns of cellular damage [1–5,16,17]. To capture these distinct molecular alterations systematically, we describe the concept of proteostasis signatures, which provides a framework for linking specific proteostasis pathway disruptions to disease mechanisms.

By characterising proteostasis signatures, we mapped proteostasis dysregulation across diseases. By defining these signatures, we aim to provide a systematic framework for understanding how proteostasis is disrupted in different disease contexts and stages. This framework has the potential to bridge gaps in our knowledge by linking specific proteostasis pathways to their functional consequences in health and disease.

## Results

### Proteostasis proteins are closely associated with disease

**Fig 1:**
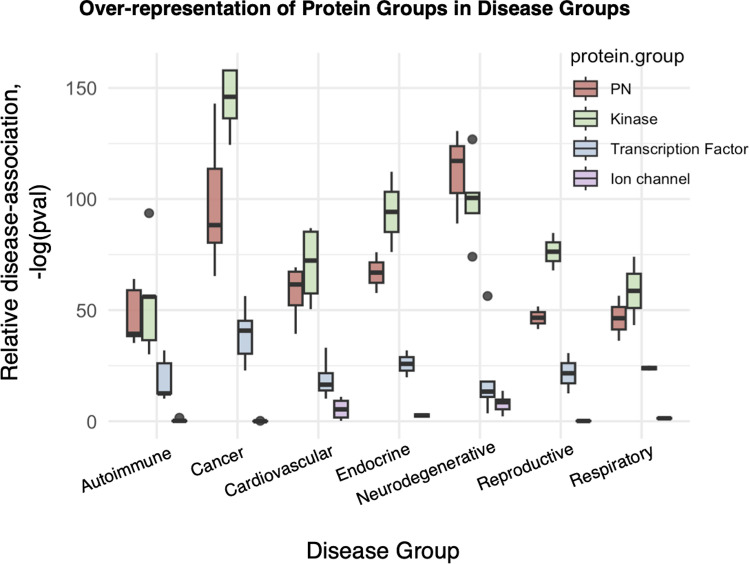
Proteostasis proteins are closely associated with disease. The relative disease association of proteostasis proteins (PN) was quantified and benchmarked against 3 control groups: kinases, transcription factors, and ion channels. Disease-association was determined by relative over-representation of a protein group within disease gene sets. This was done using the hypergeometric test measuring the statistical significance of their prevalence within each disease gene set. P-values were plotted on a -log(p-value) scale, with higher values representing stronger significance. Based on this quantification, proteostasis proteins are significantly over-represented in all the disease groups studied. They are relatively more disease associated than transcription factors, and in some cases even than kinases.

We first asked whether the proteins involved in proteostasis are preferentially associated with disease. To this end, we analysed a recent comprehensive map of the human proteostasis network [18,19] and used the proteins within the network as our reference set of proteostasis proteins. We computed their association with 32 diseases from 7 disease groups (S1 Table) by studying the prevalence of proteostasis proteins within the gene set of each disease (disease gene set). The method for generating each disease gene set is described in Materials & Methods. Our results show that proteostasis proteins are closely associated with disease, as they are significantly over-represented in disease protein sets (Fig 1). Over-representation analysis of each of the 4 protein groups within the top 500 disease-associated genes for every disease was computed with the hypergeometric test.

We then compared the disease association of the proteostasis proteins against 3 well characterized disease-associated functional protein groups: kinases, transcription factors, and ion channels. Kinases [20,21] and transcription factors [22,23] are essential regulatory proteins controlling diverse events in cellular signalling and gene transcription. They were selected as positive control groups, as they have been widely reported to be implicated in a range of diseases [20–23]. Ion channels are membrane proteins that regulate signal transduction across cell membranes [24,25], and were selected as a negative control group, as only 2 of our 7 disease groups studied (cardiovascular and neurodegenerative) are commonly associated with ion channels [24–26]. As expected, kinases and transcription factors are highly over-represented across the disease groups, while ion channels are over-represented in the neurodegenerative and cardiovascular disease groups (Fig 1).

Our analysis reveals a strong relevance of proteostasis proteins in disease, with almost comparable disease-association with kinases (Fig 1**),** which are a key targeted group of drug targets [27].

## Proteostasis profiles of disease

**Fig 2:**
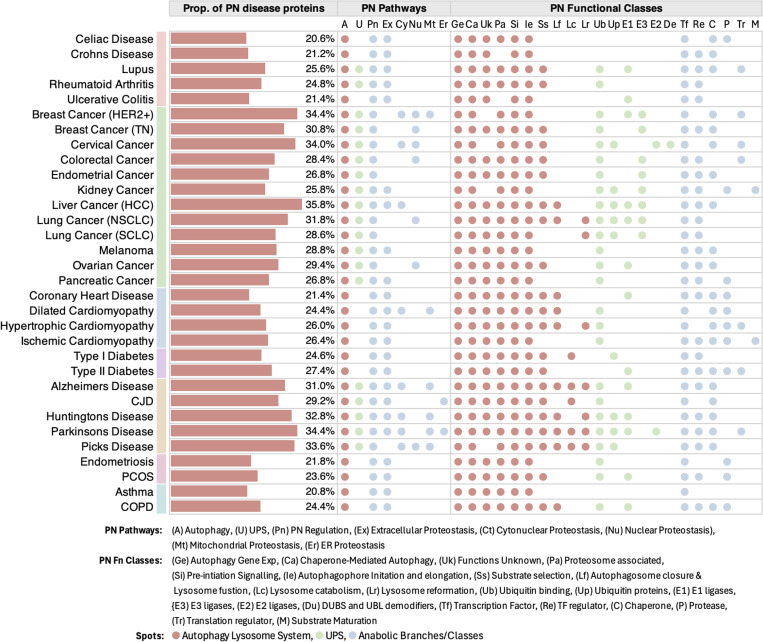
Proteostasis profiles of diseases. For each of the 32 diseases included in this study, we computed the fraction of proteostasis proteins within each disease gene set (brown bars). Across the 32 diseases, the fraction of proteostasis proteins within disease gene sets ranged from 20% to 36%. We further decomposed disease genes involved in proteostasis into their relevant proteostasis pathways and functional classes. This was done by identifying which pathway/functional class was over-represented within the proteostasis proteins corresponding to the genes of each disease gene set. The statistical significance of over-representation of a pathway or functional class was determined using the hypergeometric test (p-value < 0.01, represented as a coloured dot) and represented as a spot within the figure. Colour-coding of the spots reflect their involvement in the autophagy-lysosome pathway (brown), the ubiquitin-proteasome system (green) and the anabolic system (blue).

For each of the 32 diseases included in the study, we created a profile quantifying the proportion of proteostasis proteins within each disease gene set and associating the relevant proteostasis network pathways and functional classes to the disease (Fig 2). Based on our profiling, proteostasis proteins comprise a large portion of disease gene sets in cancer (25–36%) and neurodegenerative diseases (30–35%) (Fig 2).

At the pathway level, proteostasis proteins involved in the autophagy-lysosome pathway (ALP) and proteostasis regulation are consistently over-represented across all diseases. We found that ubiquitin-proteasome system (UPS) proteins are closely associated with cancers and neurodegenerative diseases but not with other disease groups (Fig 2). In contrast extracellular proteostasis proteins are over-represented in all disease groups except cancer (Fig 2).

At the class level, most classes of the ALP machinery appear over-represented in disease gene sets, as observed at the pathway level. We highlight: (a) UPS ubiquitin-binding proteins are significant in cancers and neurodegenerative diseases, while UPS E3 ligases are significantly represented only in cancers; (b) transcription factors, as reported extensively in the literature, are also found to be well-represented across all disease types included in the study; and (c) molecular chaperones are a functional class with strong associations with cardiovascular and neurodegenerative diseases.

## Proteostasis states in disease

**Fig 3:**
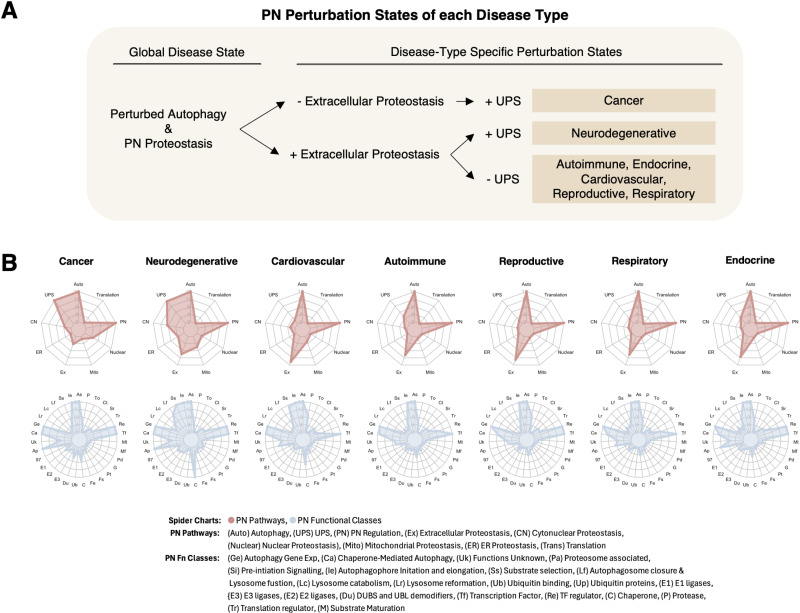
Characteristic proteostasis perturbation states and patterns in disease. (A) Three generalised proteostasis perturbation states are capable of discriminating disease types: (i) ALP + UPS + ER- (cancers), (ii) ALP + UPS + ER+ (neurodegenerative diseases), and (iii) ALP + UPS- and ER+ (other disease types analysed in this study). (B) Distinct patterns in enriched proteostasis network pathways (red spider plots) and functional classes (blue spider plots) reflect disease-relevant trends – notably, cancers and neurodegenerative diseases have distinct enrichment patterns compared to cardiovascular, autoimmune, reproductive, respiratory, and endocrine that have fairly similar patterns. The spider plots depict trends of over-representation of the relevant proteostasis network pathways and functional classes across all 7 disease types. Over-representation was determined using the hypergeometric test (p-value < 0.01).

Based on the observations from the proteostasis network profiles of the diseases, we identified 3 distinct proteostasis states in disease (Fig 3A). These proteostasis states describe disease in terms of characteristic perturbations of the proteostasis network. The most important pathways of the proteostasis network for the definition of these states are ALP, UPS and proteostasis regulation. The first proteostasis state is characterized by significant UPS perturbation but limited involvement of extracellular proteostasis (Fig 3A). This state is characteristic of cancer (Fig 3B). The second proteostasis state involves extensive perturbation of both UPS and extracellular proteostasis (Fig 3A). This state is predominantly presented in neurodegenerative diseases (Fig 3B). The third proteostasis state involves the distinctive deregulation of extracellular proteostasis but limited in UPS involvement (Fig 3A). This state is more common and less discriminatory, with autoimmune, endocrine, cardiovascular, reproductive, and respiratory diseases all presenting this trend.

### Proteostasis signatures of disease

**Fig 4:**
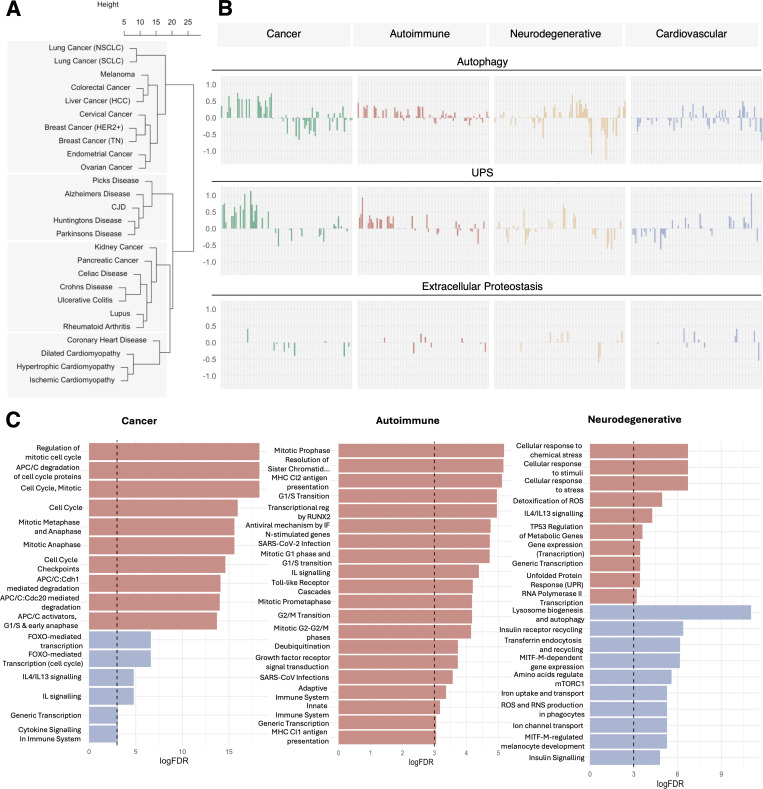
Proteostasis signatures of disease. (A) Unsupervised clustering of cancers, neurodegenerative diseases, autoimmune diseases, and cardiovascular disease resulted in 4 clusters. The clusters were mostly by disease type, with the notable exception of pancreatic cancer and kidney cancer clustering with autoimmune diseases. (B) Proteostasis signature trends reveal that cancers and autoimmune diseases have a large proportion of common genes perturbed in similar patterns. In contrast, neurodegenerative diseases are perturbed in opposite directions. Each bar represents a gene from the relevant proteostasis network pathway. (C) Functional implications of the proteostasis signatures. The top enriched pathways (up to 10 each) for upregulated (red bars) and downregulated (blue bars) genes for each cluster type are shown.

To study the perturbation of the proteostasis network in disease and identify generalizable gene-wise disease signatures for characterizing disease types, we defined the disease-specific proteostasis signatures. We first considered 4 groups of disorders with distinct proteostasis states: cancer, neurodegenerative diseases, autoimmune diseases, and cardiovascular diseases. Clustering these diseases based on their disease-associated genes resulted in 4 clusters. The diseases clustered largely according to their proteostasis states, with a notable exception, in which kidney cancer and pancreatic cancer clustered with autoimmune diseases (Fig 4A), a finding consistent with the bidirectional association between cancer and autoimmune disorders reported in the literature [28,29]. Extracting generalized gene-wise disease signatures for ALP, UPS, and extracellular proteostasis identified similar proteostasis signatures of cancers and autoimmune disorders, indicating that similar genes are perturbed in related directions (Fig 4B). In addition, we found that proteostasis proteins associated with neurodegenerative diseases are often perturbed in opposite directions to cancers and autoimmune diseases (Fig 4B).

To gain insight into the functional pathways implicated due to proteostasis alteration in disease, we carried out pathway enrichment analysis. Our results revealed disease pathways modulated by proteostasis (Fig 4C). In cancer, upregulated proteostasis proteins were found to be enriched in cell cycle pathways; in autoimmune diseases, cell cycle activation was also observed along with hyperactivation of the innate immune system; in neurodegenerative diseases, the unfolded protein response (UPR) was seen to be activated, likely as a natural reaction to the accumulation of aggregation-prone and damaged proteins. Other dysregulated pathways in neurodegenerative diseases included those involved in protein clearance, such as lysosome formation, autophagy, endocytosis, and recycling, alongside MITF-M-regulated pathways critical for maintaining brain function.

### Proteostasis perturbations in disease onset and progression

**Fig 5:**
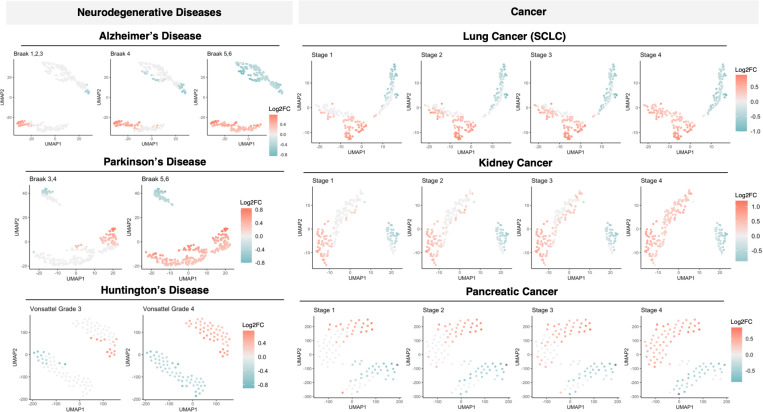
Temporal progression of proteostasis perturbations across disease types. Patient samples from each disease was compared against against healthy controls. Differential gene analysis reveals that perturbation of the proteostasis network (PN) occurred progressively in neurodegenerative diseases but early in cancers. Each point represents a gene significantly perturbed in disease compared to controls, coloured by its direction and magnitude of change in disease conditions.

Next, we studied proteostasis perturbations on a temporal scale over the course of disease staging in 6 diseases (3 neurodegenerative diseases and 3 cancers) for which staging data are available: Alzheimer’s disease (AD), Parkinson’s disease (PD), Huntington’s disease (HTT), lung cancer, kidney cancer, and pancreatic cancer (Fig 5). For each disease, differential gene expression analysis was carried out by disease stage against healthy controls. Our results reveal that proteostasis perturbations, regardless of upregulation or downregulation, occur progressively in neurodegenerative diseases but early in cancers (Fig 5). These trends were conserved across all diseases included in this analysis for both disease types.

**Fig 6:**
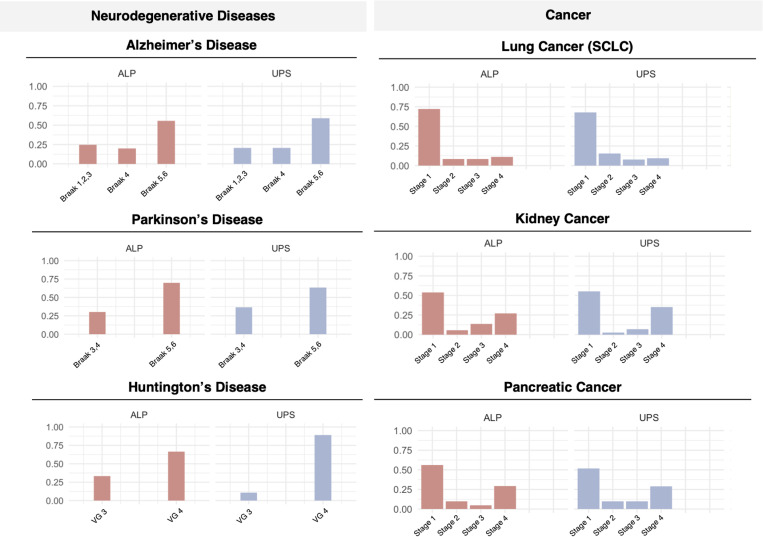
Temporal patterns of ALP and UPS perturbations in the progression of cancer and neurodegenerative diseases. The proportion of the ALP and UPS genes affected in each stage of disease is calculated and depicted. While ALP and UPS perturbations are indicative of disease states in both cancers and neurodegenerative diseases, a large proportion of ALP and UPS genes affected only in later stages of disease compared to the early implication of these genes in early stages of cancers.

Further findings from this disease progression analysis revealed that although both ALP and UPS perturbations are indicative of disease states in both neurodegenerative diseases and cancer, they occur at different stages of disease progression. A larger proportion of affected ALP and UPS proteins are perturbed in early stages of the 3 cancers studied, but only at late stages for the 3 neurodegenerative diseases studied (Fig 6). This result is in line with existing observations that damaged proteins are accumulated in neurodegenerative diseases over the course of aging that leads to toxicity, while cancer cells hijack the proteostasis network to enable survival and proliferation. We also examined genes affected in early-stages (Braak 1/2) of AD (S1 Fig). Our workflow enables the identification of previously reported early-stage AD genes such as YAP1 [30].

**Fig 7:**
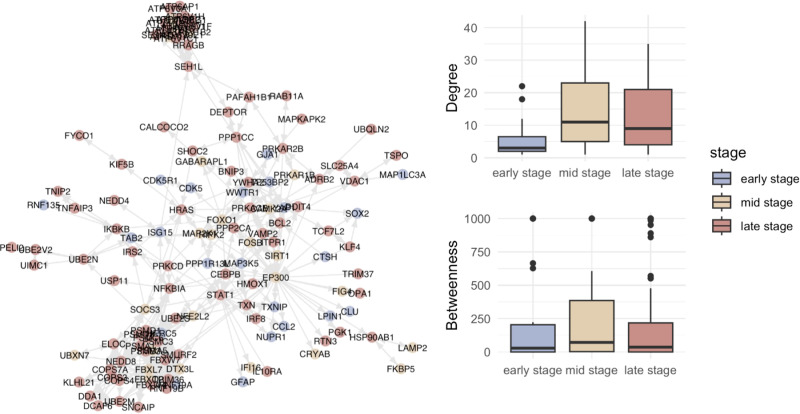
Central role of mid-stage regulatory proteins in the proteostasis network perturbations in AD. The functional interaction network of AD-associated proteostasis network genes (from all disease stages) are shown. Genes affected in the early stage are depicted as blue nodes; mid stage as beige nodes; and late stage as red nodes. A stage-wise quantification of the degree and betweenness centralities of the AD-associated proteostasis network genes within the network presented reveals that proteostasis proteins perturbed in mid-stage AD (Braak 2/3) are most central in the AD proteostasis network. The degree centrality measures the number of connections of a protein within the network, and the betweenness measures the extent to which a protein lies on the shortest path between protein pairs within the network. Proteins with high degree and betweenness are likely to play key regulatory roles in their functional networks. The upper and lower bounds of the boxplots represent the interquartile range of degree/betweenness for genes associated with each disease stage. The line contained in the box represents the 50^th^ percentile of degree/betweenness for genes associated with each disease stage. Whiskers represent non-outlying extreme points while data points beyond the whiskers are plotted individually.

We further studied how proteostasis perturbations spread across disease progression. We hypothesized that the proteostasis proteins perturbed at earlier stages are central regulators of the proteostasis network, resulting in downstream disarray. To test this possibility, we mapped the perturbation of the proteostasis network in AD (Fig 7). We then quantified and compared the degree and betweenness centrality of the proteostasis proteins involved in each stage. The degree measures the number of connections a protein within the network, and betweenness measures the extent to which a protein lies on the shortest path between protein pairs within the network. Proteins with high degree and betweenness are likely to play key regulatory roles in their functional networks. Based on these metrics, we found that, unlike our initial hypothesis, proteostasis proteins perturbed in mid-stage AD (Braak 2/3) are most central in the AD proteostasis network (Fig 7). This result prompts further investigations on whether early-stage AD proteostasis proteins could be seeds that affect regulatory proteins contributing to proteostasis collapse in later stages.

### Proteostasis perturbations due to smoking are associated with disease risk

Given that proteostasis perturbations can be observed at early stages of disease, we further investigate if disease risk factors alter the proteostasis network promoting disease susceptibility. Many existing studies have shown that the proteostasis network is altered with age, which is a major risk factor for many chronic diseases. By building on these results, we extend our analysis to smoking, another key risk factor for many chronic diseases that may increase disease risk via the proteostasis network. Smoking is particularly interesting because it has been reported to increase risk for some diseases while decreasing risk for others. Smoking is a risk factor for the development of chronic obstructive pulmonary disease (COPD) [31,32], lung cancer [33], breast cancer [34,35], and coronary heart disease [36,37]. However, it has also been reported to reduce the risk for ulcerative colitis [38,39], endometrial cancer [40–42], endometriosis [43–45], and PD [46–48].

**Fig 8:**
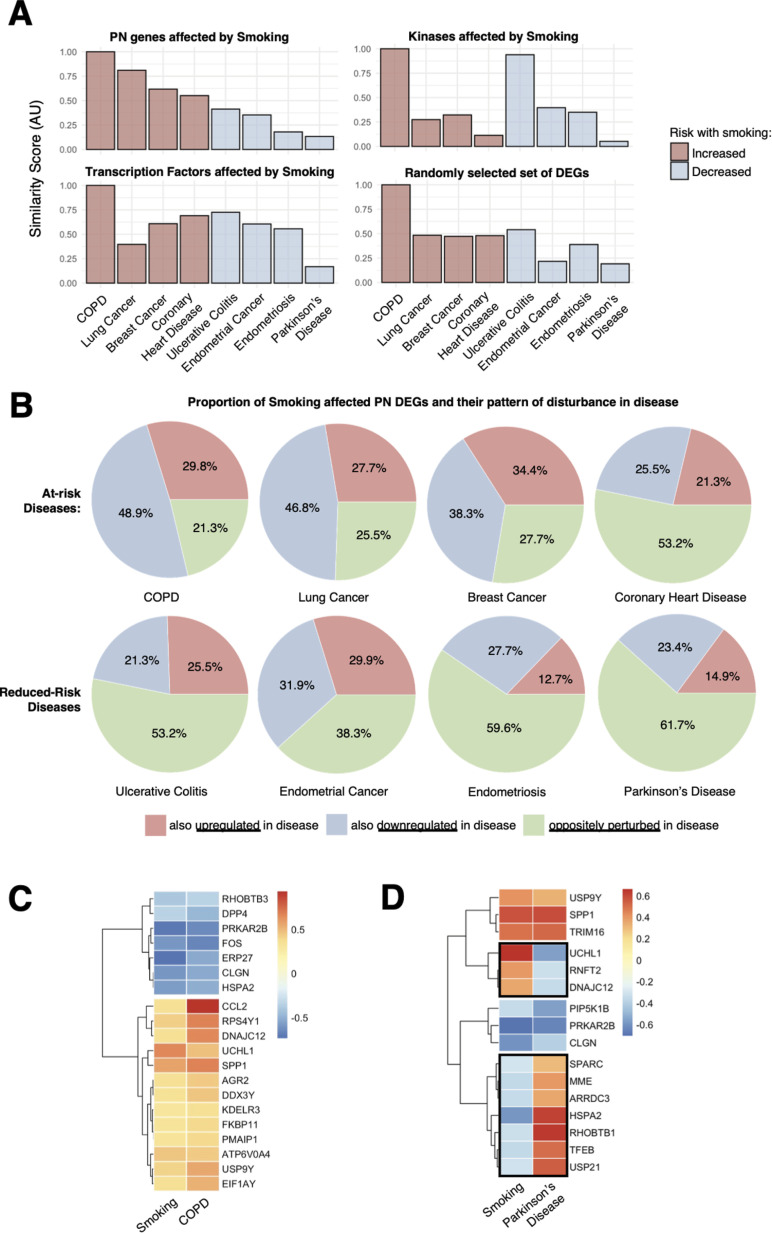
Proteostasis perturbations due to smoking are indicative of disease risk. (A) Smokers present a higher similarity of proteostasis perturbations with at-risk diseases compared to reduced-risk diseases. Computing similarities of proteostasis proteins is more indicative of disease risk as compared to smoking-impacted kinases, transcription factors, or a random sample of differentially expressed genes. (B) At-risk diseases have a higher directional similarity of their perturbed proteostasis proteins with smoking. In contrast, reduced-risk diseases have a large proportion of perturbed proteostasis proteins that are deregulated in the opposite direction. (C) Genes encoding proteostasis proteins are perturbed similarly in smokers and patients with COPD. Genes similarly perturbed between smoking and COPD are likely to be contributive toward increasing COPD risk and onset. (D) Proteostasis proteins corresponding to genes perturbed in smokers and patients with PD. Proteostasis proteins oppositely perturbed between smoking and PD are likely to be protective against PD.

The differentially expressed genes in smokers were compared against 8 disease gene sets: COPD, lung cancer, breast cancer, coronary heart disease, ulcerative colitis, endometrial cancer, endometriosis, and PD. The differentially expressed genes in smokers were obtained by carrying out differential gene expression analysis between smokers and non-smokers with no reported diseases. According to our hypothesis, we expected to find a higher similarity in perturbed proteostasis genes in smokers with diseases with increased risk due to smoking (hereafter referred to as ‘at-risk’ diseases), and a lower level of similarity between smokers and diseases with lowered risk due to smoking (hereafter referred to as ‘reduced-risk’ diseases). Based on our results, we find that proteostasis perturbations are indeed more similar between smokers and patients with at-risk diseases, and less similar between smokers and patients with reduced-risk diseases (Fig 8A). The Jaccard index was used to quantify similarity between perturbed proteostasis proteins due to smoking and proteostasis proteins within each disease gene set (Materials and Methods), then normalized for plotting in Fig 8A. We found that quantifying similarities between smoking-impacted proteostasis was more indicative of risk of disease than smoking-impacted kinases or transcription factors (Fig 8A), both of which are protein groups strongly associated with disease, as discussed earlier. We then compared the pairwise directional similarity of proteostasis perturbation between the overlapping smoking-perturbed proteostasis and disease-associated proteostasis perturbations. Our comparison revealed that smoking results in a larger directional similarity (i.e., proteostasis genes upregulated due to smoking, are also upregulated in disease, and proteostasis genes downregulated due to smoking, are also downregulated in disease) with at-risk diseases vis-à-vis reduced-risk diseases that have a large proportion of their proteostasis being perturbed in opposite directions (Fig 8B).

These observations suggest that the proteostasis similarly perturbed between smoking and at-risk diseases are likely to contribute toward increasing disease risk and the development of disease pathologies. Given this observation, we further investigated the proteostasis proteins perturbed similarly in smokers and patients with COPD (at-risk disease with highest similarity). For example, CCL2, an extensively studied protein target in COPD [49,50], is upregulated at both the transcriptomic (Fig 8C) and proteomic level [51] due to smoking. Similarly, proteostasis oppositely perturbed between smoking and reduced-risk diseases are likely to be protective against disease development. Hence, we investigated PD (reduced-risk disease with largest dissimilarity) in more detail. Our analysis identifies UCHL1 to be over-expressed in smokers (Fig 8D), mirroring earlier reports of smoking-dependent upregulation at the proteomic level [52]. This upregulation directly contrasts the downregulation of the UCHL1 gene observed in PD patients (Fig 8D), which correlates with the decreased risk for PD found in smokers. Given that UCHL1 has been reported to be a susceptibility gene for PD and proposed as a potential target for therapy [52], whose downregulation contributes to protein aggregation in Lewy bodies [53] – a hallmark of PD pathology, it is possible that the upregulation of UCHL1 due to smoking protects against UCHL1 loss-of-function that predisposes the cells to PD related symptoms. Moving forward, it will be interesting to further explore the other genes (Fig 6C,D) that present this trend to uncover key contributors to disease vulnerability thus supporting efforts in preventive care.

### Discussion

In this work, we quantified the involvement of the proteostasis network across diseases. We thus found specific disease signatures that characterise disruptions in protein homeostasis in various diseases. Upon further analysis, we found temporal patterns of proteostasis network involvement across disease development that differentiate similar static presentations of proteostasis network disturbances in disease states. In addition, we uncovered how risk factors such as smoking greatly impact the proteostasis network, likely priming cells with increased vulnerability for disease environments. We contextualise our findings within the current literature in the following.

Based on our profiling of proteostasis network functions associated with 32 diseases, we proposed 3 generalised proteostasis network disease perturbation states: (i) ALP + UPS + ER- (cancers), (ii) ALP + UPS + ER+ (neurodegenerative diseases), and (iii) ALP + UPS- and ER+ (other disease types analysed in this study). From this analysis, we observed that perturbations in autophagy/ALP represent a general state of disease. This finding is consistent with the widespread implication of ALP in many disease types [54–58], and studied extensively in cancer [59], neurodegenerative diseases [60–62], autoimmune diseases [57,63–67], respiratory diseases [68–70], cardiovascular diseases [71–74], and endocrine disorders [75–78]. Similarly, the UPS has been widely reviewed in its links to cancer and neurodegenerative diseases [79–84]. Studies have explored the targeting of UPS to modulate autoimmune diseases [85,86] and investigated the relevance of UPS in other disease groups such as cardiovascular diseases [87] and diabetes [88,89], albeit much less extensively. Furthermore, perturbations in extracellular proteostasis have been reported in disease types such as cardiovascular diseases [90], as also observed in our analysis, but also more recently discussed in the context of the extracellular matrix in cancer [91], not identified as a key proteostasis network signature. Perturbation of the UPS being identified as a representative disease signature for cancers and neurodegenerative diseases, or extracellular proteostasis network for non-cancer diseases, within our analysis but not the other disease types explored in literature may be the result of methodological processes, as for example the generation of the disease-gene sets (using the top 500 most associated diseases) and/or p-value cut-offs (we used strict p-values of 0.01). Alternatively, the analysis may also suggest the possibility that while multiple proteostasis network processes may play a role in altering disease states, some processes might have a stronger signal than others hence showing up upon filtering for top disease-associated genes and stricter p-values. Indeed, a comparison against alternative disease-gene sets or generating a consensus gene set from multiple established sources such as DisGeNET [92] or COSMIC (https://cancer.sanger.ac.uk/cosmic) for cancer can help establish the robustness of the identified proteostasis network disease signatures and disease states.

Deconvolution of the static disease states over progression revealed differences in proteostasis network disturbances over the course of disease. We reported that ALP and UPS are implicated early in disease for all studied cancers, while it is progressively changed in neurodegenerative diseases. This finding is supported by reports describing the role of autophagy in tumour initiation in cancer, an early study identified the contribution of decreased Beclin 1 levels (an autophagy regulator) in tumorigenesis [93], followed by multiple reviews studying the mechanisms of autophagy in the initial stages of cancer [94–97] and later in metastasis [98–102]. Like ALP, UPS has also been reported to play a role in tumorigenesis in early cancer stages [103,104]. Components of the UPS have been identified as potential targets for cancer therapies, including bortezomib [105], carfilzomib [106], b-AP15 [107], and VLX1570 [108] (proteasome inhibitors), pevonedistat [109] (E1 ligase inhibitor), and the nutlins [110] (E3 ligase inhibitors). In contrast, neurodegenerative diseases are characterised by a gradual build-up of misfolded proteins and their aggregates, as well as a progressive loss of neurons. The decline of proteostasis functions with time such as in aging is a likely contributor to this phenomenon. This has been exemplified in a study that found the progressive decrease in cellular proteostasis contributes to ALS onset [111].

An extension of the workflow in this study toward small patient cohorts would also be a unique application worth exploring. In this study, we illustrated the potential of identifying proteostasis network genes affected in early-stage AD, which is often characterised by small patient cohorts as it is difficult to identify and diagnose AD at early stages. In a similar way, this workflow can in principle be applied toward smaller patient cohorts for example rare diseases. However, such workflows may still be limited in effectiveness for some small datasets such as single/paired samples.

Finally, we established a link between smoking and disease states via the proteostasis network, showing that smoking affects proteostasis network components in a similar way to that in smoking-risk diseases. This result is supported by previous studies showing that inhaled smoke can impair protein folding resulting in ER stress [112] and smoke-induced aggresome formation contributes to COPD [113], amongst others. We note that other risk factors for disease, such as obesity can also have significant impacts on the proteostasis network promoting disease vulnerability whereby loss of proteostasis due to obesity leads to cardiovascular disease [114] or hypothalamic dysfunction [115]. Given these observations, further exploration of risk factors may allow us to uncover mechanisms that raise disease susceptibility allowing for strengthening preventive efforts.

We highlight that caution must be exercised when interpreting these signatures as association does not necessarily imply causation. Extensive effort needs to go into validating these mechanisms to develop robust and effective interventions. At present, disease signature studies are still predominantly research-based due to limitations like the interpretability of the disease signatures themselves. In addition, the disease signatures do not always have a one-to-one mapping with disease aetiologies, as for example stress-response patterns often overlap across diseases, or the clustering of pancreatic and kidney cancer with autoimmune diseases. These multiple mappings make assigning proteostasis disruptions to specific diseases extremely complex, and so that fitting diseases into known signatures could lead to incorrect conclusions. Given this complexity, differentiation of proteostasis network disease patterns needs to be done rigorously and interpreted accordingly to avoid unreliable associations.

## Materials and Methods

### Proteostasis network

A comprehensive list of proteostasis network components [18,19] was obtained from the Proteostasis Consortium (https://www.proteostasisconsortium.com/).

### Disease gene sets

The top 500 genes for each disease, based on PandaOmics [116], were determined to be disease associated and made up the gene set. This ranking was derived by comparing disease samples to tissue-matched controls in PandaOmics v2.0. The final scores used for ranking are a result of aggregating multiple omics inputs for each gene using a neural network. The multi-omics input include: (i) mRNA expression (level of differential gene expression in disease versus control), (ii) interactome community (the density of known targets, disease-related genes, and differentially expressed genes in its protein-protein interaction network), (iii) causal inference (estimating the number of genes regulated by similar transcription factors), (iv) overexpression (characterizing the effects of gene knock-in/knock-out on cell lines), (v) mutated disease sub-modules (assessing gene relevance based on OMIM, ClinVar, and Open Targets data), (vi) mutations (a combined score from genome and transcriptome-wide association studies), (vii) pathway analysis (evaluating a gene’s role in Reactome pathways using iPANDA and transcriptomic data), (viii) network neighbors (based on the number of directly connected differentially expressed genes in the protein interaction network), and (ix) disease relevance (aggregated scores from OMIM, ClinVar, and Open Targets). All patient datasets used in this study are documented in S2 Table. The proteostasis subset of each disease gene set was obtained by finding the intersection of our full list of proteostasis components with each disease gene set. All disease gene sets are available in S3 Table.

### Over-representation analysis of proteostasis proteins

A list of kinases, transcription factors, and ion channels (S4 Table) was obtained from the PandaOmics database that annotated more than 20,000 proteins. Over-representation analysis was carried out using the hypergeometric test that measures the statistical significance of each group of proteins being over-represented in the disease gene set. P-values were plotted on a -log(p-value) scale, with higher values representing stronger significance.

### Over-representation analysis of proteostasis network pathways and functional classes

The hypergeometric test was used to quantify the enrichment of every pathway/functional class within the disease gene set for each disease. A cutoff of p-value <0.01 was used in the profiling presented in Fig 5.

### Unsupervised clustering of diseases

The Jaccard Index was used to calculate similarity scores between diseases. This similarity matrix was used for hierarchical clustering (hclust function in R) which generated 4 clusters (Fig 6A).

### Pathway enrichment analysis

Pathway enrichment analysis was carried out by calculating the probability of a disease gene set being over-represented in a pathway. The Reactome pathways were used for this analysis. A cut-off of false discovery rates (FDR) < 0.05 and more than 2 genes per pathway were applied. For plotting, the most upregulated and downregulated (up to 10 each) were included.

### Disease staging transcriptomic datasets

Only diseases for which transcriptomic datasets with disease staging information that was readily available were included in this study. The datasets used were: GSE48350 and GSE84422 (AD); GSE49036 and GSE42966 (PD); GSE64810 and GSE79666 (HTT); GSE30219 (lung cancer); GSE53757, GSE76207, and GSE126964 (kidney cancer); GSE62452 (pancreatic cancer).

### Smoking transcriptomic datasets

Datasets that included samples from healthy controls that were smokers and non-smokers were obtained to study the impact of smoking on the proteostasis network. Datasets obtained and used include GSE22047 and GSE108134.

### Differential expression analysis

Differential expression analysis was used to quantify expression differences between conditions: disease stage vs healthy control, or smokers vs non-smokers, for disease staging and smoking transcriptomic datasets respectively. All analyses were performed using the Limma method [117].

### Similarity scores

The Jaccard index was used to calculate similarity scores. It measures the ratio of the overlap between the perturbed proteostasis genes due to smoking and the proteostasis genes within each disease gene set to their union.

### Illustration creation

The proteostasis profiles of diseases (Fig 2) were created in Excel. All other plots were created in R using the ggplot2 package.

## Supporting information

S1 Fig PN genes implicated at early-stages of AD (braak 1/2). Red indicates upregulation of a PN gene compared to control. Blue indicates downregulation of a PN gene compared to control. Intensity depicts extent of log2foldchange.
(PNG)

S1 Table List of diseases and their corresponding disease groups. (XLSX)

S2 Table Transcriptomic datasets used for comparing diseases vs control patient data. (XLSX)

S3 Table Disease gene sets for all 32 diseases studied. (XLSX)

S4 Table List of kinases, transcription factors, and ion channels used as controls. (XLSX)

## References

[1] Hipp MS, Kasturi P, Hartl FU. The proteostasis network and its decline in ageing. Nat Rev Mol Cell Biol. 2019;20(7):421–35. doi: 10.1038/s41580-019-0101-y 30733602

[2] Labbadia J, Morimoto RI. The biology of proteostasis in aging and disease. Annu Rev Biochem. 2015;84:435–64. 25784053 10.1146/annurev-biochem-060614-033955 PMC4539002

[3] Morimoto RI, Cuervo AM. Proteostasis and the aging proteome in health and disease. J Gerontol A Biol Sci Med Sc. 2014;69:S33–8. 10.1093/gerona/glu049 PMC4022129 24833584

[4] Balch WE, Morimoto RI, Dillin A, Kelly JW. Adapting proteostasis for disease intervention. Science. 2008;319(5865):916–9. doi: 10.1126/science.1141448 18276881

[5] Klaips CL, Jayaraj GG, Hartl FU. Pathways of cellular proteostasis in aging and disease. J Cell Biol. 2018;217(1):51–63. doi: 10.1083/jcb.201709072 29127110 PMC5748993

[6] Wilson MR, Satapathy S, Vendruscolo M. Extracellular protein homeostasis in neurodegenerative diseases. Nat Rev Neurol. 2023;19(4):235–45. doi: 10.1038/s41582-023-00786-2 36828943

[7] Knowles TPJ, Vendruscolo M, Dobson CM. The amyloid state and its association with protein misfolding diseases. Nat Rev Mol Cell Biol. 2014;15(6):384–96. doi: 10.1038/nrm3810 24854788

[8] López-Otín C, Blasco MA, Partridge L, Serrano M, Kroemer G. The hallmarks of aging. Cell. 2013;153(6):1194–217. doi: 10.1016/j.cell.2013.05.039 23746838 PMC3836174

[9] Ciocca DR, Clark GM, Tandon AK, Fuqua SA, Welch WJ, McGuire WL. Heat shock protein hsp70 in patients with axillary lymph node-negative breast cancer: prognostic implications. J Natl Cancer Inst. 1993;85(7):570–4. doi: 10.1093/jnci/85.7.570 8455204

[10] Cappello F, David S, Rappa F, Bucchieri F, Marasà L, Bartolotta TE, et al. The expression of HSP60 and HSP10 in large bowel carcinomas with lymph node metastase. BMC Cancer. 2005;5:139. doi: 10.1186/1471-2407-5-139 16253146 PMC1289279

[11] Lazaris AC, Chatzigianni EB, Panoussopoulos D, Tzimas GN, Davaris PS, Golematis BC. Proliferating cell nuclear antigen and heat shock protein 70 immunolocalization in invasive ductal breast cancer not otherwise specified. Breast Cancer Res Treat. 1997;43(1):43–51. doi: 10.1023/a:1005706110275 9065598

[12] Nanbu K, Konishi I, Komatsu T, Mandai M, Yamamoto S, Kuroda H, et al. Expression of heat shock proteins HSP70 and HSP90 in endometrial carcinomas. Correlation with clinicopathology, sex steroid receptor status, and p53 protein expression. Cancer. 1996;77(2):330–8. doi: 10.1002/(SICI)1097-0142(19960115)77:2<330::AID-CNCR16>3.0.CO;2-2 8625242

[13] Lawrence MS, Stojanov P, Polak P, Kryukov GV, Cibulskis K, Sivachenko A, et al. Mutational heterogeneity in cancer and the search for new cancer-associated genes. Nature. 2013;499(7457):214–8. doi: 10.1038/nature12213 23770567 PMC3919509

[14] Alexandrov LB, Nik-Zainal S, Wedge DC, Aparicio SAJR, Behjati S, Biankin AV, et al. Signatures of mutational processes in human cancer. Nature. 2013;500(7463):415–21. doi: 10.1038/nature12477 23945592 PMC3776390

[15] Kandoth C, McLellan MD, Vandin F, Ye K, Niu B, Lu C, et al. Mutational landscape and significance across 12 major cancer types. Nature. 2013;502(7471):333–9. doi: 10.1038/nature12634 24132290 PMC3927368

[16] Hetz C, Glimcher LH. Protein homeostasis networks in physiology and disease. Curr Opin Struct Biol. 2011;23:123. 10.1016/j.ceb.2011.01.004 PMC3078169 21306885

[17] Wen JH, He XH, Feng ZS, Li DY, Tang JX, Liu HF. Cellular protein aggregates: formation, biological effects, and ways of elimination. Int J Mol Sci. 2023;24:8593. 37239937 10.3390/ijms24108593 PMC10217863

[18] Elsasser S, Elia LP, Morimoto RI, Powers ET, Finley D, Costa B, et al. A comprehensive enumeration of the human proteostasis network. 2. Components of the autophagy-lysosome pathway. bioRxiv. 2023. doi: 10.1101/2023.1103.1122.533675

[19] Elsasser S, Elia LP, Morimoto RI, Powers ET, Harvard Medical School group (analysis), Finley D, University of California, San Francisco and Gladstone Institutes group I, et al. A comprehensive enumeration of the human proteostasis network. 1. Components of translation, protein folding, and organelle-specific systems. bioRxiv. 2022. doi: 10.1101/2022.1108.1130.505920

[20] Manning G, Whyte DB, Martinez R, Hunter T, Sudarsanam S. The protein kinase complement of the human genome. Science. 2002;298(5600):1912–34. doi: 10.1126/science.1075762 12471243

[21] Klaeger S, Heinzlmeir S, Wilhelm M, Polzer H, Vick B, Koenig P-A, et al. The target landscape of clinical kinase drugs. Science. 2017;358(6367):eaan4368. doi: 10.1126/science.aan4368 29191878 PMC6542668

[22] Lambert SA, Jolma A, Campitelli LF, Das PK, Yin Y, Albu M, et al. The Human Transcription Factors. Cell. 2018;172(4):650–65. doi: 10.1016/j.cell.2018.01.029 29425488 PMC12908702

[23] Vaquerizas JM, Kummerfeld SK, Teichmann SA, Luscombe NM. A census of human transcription factors: function, expression and evolution. Nat Rev Genet. 2009;10(4):252–63. doi: 10.1038/nrg2538 19274049

[24] Hübner CA, Jentsch TJ. Ion channel diseases. Hum Mol Genet. 2002;11(20):2435–45. doi: 10.1093/hmg/11.20.2435 12351579

[25] Jentsch TJ, Hübner CA, Fuhrmann JC. Ion channels: function unravelled by dysfunction. Nat Cell Biol. 2004;6(11):1039–47. doi: 10.1038/ncb1104-1039 15516997

[26] Niemeyer BA, Mery L, Zawar C, Suckow A, Monje F, Pardo LA, et al. Ion channels in health and disease. 83rd Boehringer Ingelheim Fonds International Titisee Conference. EMBO Rep. 2001;2(7):568–73. doi: 10.1093/embo-reports/kve145 11463739 PMC1083959

[27] Bhullar KS, Lagarón NO, McGowan EM, Parmar I, Jha A, Hubbard BP, et al. Kinase-targeted cancer therapies: progress, challenges and future directions. Mol Cancer. 2018;17(1):48. doi: 10.1186/s12943-018-0804-2 29455673 PMC5817855

[28] Giat E, Ehrenfeld M, Shoenfeld Y. Cancer and autoimmune diseases. Autoimmun Rev. 2017;16(10):1049–57. doi: 10.1016/j.autrev.2017.07.022 28778707

[29] Sakowska J, Arcimowicz Ł, Jankowiak M, Papak I, Markiewicz A, Dziubek K, et al. Autoimmunity and Cancer-Two Sides of the Same Coin. Front Immunol. 2022;13:793234. doi: 10.3389/fimmu.2022.793234 35634292 PMC9140757

[30] Tanaka H, Homma H, Fujita K, Kondo K, Yamada S, Jin X, et al. YAP-dependent necrosis occurs in early stages of Alzheimer’s disease and regulates mouse model pathology. Nat Commun. 2020;11(1):507. doi: 10.1038/s41467-020-14353-6 31980612 PMC6981281

[31] Chung C, Lee KN, Han K, Shin DW, Lee SW. Effect of smoking on the development of chronic obstructive pulmonary disease in young individuals: a nationwide cohort study. Front Med (Lausanne). 2023;10:1190885. doi: 10.3389/fmed.2023.1190885 37593403 PMC10428618

[32] Laniado-Laborín R. Smoking and chronic obstructive pulmonary disease (COPD). Parallel epidemics of the 21 century. Int J Environ Res Public Health. 2009;6(1):209–24. doi: 10.3390/ijerph6010209 19440278 PMC2672326

[33] Walser T, Cui X, Yanagawa J, Lee JM, Heinrich E, Lee G, et al. Smoking and lung cancer: the role of inflammation. Proc Natl Acad Sci USA. 2008;5:811–5. 10.1513/pats.200809-100TH PMC4080902 19017734

[34] Macacu A, Autier P, Boniol M, Boyle P. Active and passive smoking and risk of breast cancer: a meta-analysis. Breast Cancer Res Treat. 2015;154(2):213–24. doi: 10.1007/s10549-015-3628-4 26546245

[35] International Agency for Research on Cancer. Painting, firefighting, and shiftwork. IARC monographs on the evaluation of carcinogenic risks to humans. 2012. PMC4781497 21381544

[36] Gallucci G, Tartarone A, Lerose R, Lalinga AV, Capobianco AM. Cardiovascular risk of smoking and benefits of smoking cessation. J Thorac Dis. 2020;12:3866. 32802468 10.21037/jtd.2020.02.47 PMC7399440

[37] Hackshaw A, Morris JK, Boniface S, Tang J-L, Milenković D. Low cigarette consumption and risk of coronary heart disease and stroke: meta-analysis of 141 cohort studies in 55 study reports. Br Med J. 2018;360. 10.1136/bmj.j5855 PMC5781309 29367388

[38] Bastida G, Beltrán B. Ulcerative colitis in smokers, non-smokers and ex-smokers. World J Gastroenterol. 2011;17(22):2740–7. doi: 10.3748/wjg.v17.i22.2740 21734782 PMC3122262

[39] Guslandi M. Nicotine treatment for ulcerative colitis. Br J Clin Pharmacol. 1999;48(4):481–4. doi: 10.1046/j.1365-2125.1999.00039.x 10583016 PMC2014383

[40] Felix AS, Yang HP, Gierach GL, Park Y, Brinton LA. Cigarette smoking and endometrial carcinoma risk: the role of effect modification and tumor heterogeneity. Cancer Causes Control. 2014;25(4):479–89. doi: 10.1007/s10552-014-0350-1 24487725 PMC4151521

[41] Zhou B, et al. Cigarette smoking and the risk of endometrial cancer: a meta-analysis. Am J Med. 2008;121:501–8. doi: 10.1016/j.amjmed.2008.01.001 18501231

[42] van den Berg M, van Duursen MB. Mechanistic considerations for reduced endometrial cancer risk by smoking. Curr Op Toxicol. 2019;14:52–9.

[43] Calhaz-Jorge C, Mol BW, Nunes J, Costa AP. Clinical predictive factors for endometriosis in a Portuguese infertile population. Hum Reprod. 2004;19(9):2126–31. doi: 10.1093/humrep/deh374 15229202

[44] Cramer DW, Wilson E, Stillman RJ, Berger MJ, Belisle S, Schiff I, et al. The relation of endometriosis to menstrual characteristics, smoking, and exercise. JAMA. 1986;255(14):1904–8. doi: 10.1001/jama.1986.03370140102032 3951117

[45] Baron JA, La Vecchia C, Levi F. The antiestrogenic effect of cigarette smoking in women. Am J Obstet Gynecol. 1990;162(2):502–14. doi: 10.1016/0002-9378(90)90420-c 2178432

[46] Mappin-Kasirer B, Pan H, Lewington S, Kizza J, Gray R, Clarke R, et al. Tobacco smoking and the risk of Parkinson disease: A 65-year follow-up of 30,000 male British doctors. Neurology. 2020;94(20):e2132–8. doi: 10.1212/WNL.0000000000009437 32371450 PMC7526668

[47] Martyn C, Gale C. Tobacco, coffee, and Parkinson’s disease: caffeine and nicotine may improve the health of dopaminergic systems. BMJ. 2003;561–2. 10.1136/bmj.326.7389.561 PMC1125458 12637374

[48] Rose KN, Schwarzschild MA, Gomperts SN. Clearing the Smoke: What Protects Smokers from Parkinson’s Disease?. Mov Disord. 2024;39(2):267–72. doi: 10.1002/mds.29707 38226487 PMC10923097

[49] Henrot P, Prevel R, Berger P, Dupin I. Chemokines in COPD: From Implication to Therapeutic Use. Int J Mol Sci. 2019;20(11):2785. doi: 10.3390/ijms20112785 31174392 PMC6600384

[50] Dong Y, Dong Y, Zhu C, Yang L, Wang H, Li J, et al. Targeting CCL2-CCR2 signaling pathway alleviates macrophage dysfunction in COPD via PI3K-AKT axis. Cell Commun Signal. 2024;22:364. 39014433 10.1186/s12964-024-01746-z PMC11253350

[51] Giebe S, Brux M, Hofmann A, Lowe F, Breheny D, Morawietz H, et al. Comparative study of the effects of cigarette smoke versus next-generation tobacco and nicotine product extracts on inflammatory biomarkers of human monocytes. Pflugers Arch. 2023;475(7):823–33. doi: 10.1007/s00424-023-02809-9 37081240 PMC10264276

[52] Carolan BJ, Heguy A, Harvey B-G, Leopold PL, Ferris B, Crystal RG. Up-regulation of expression of the ubiquitin carboxyl-terminal hydrolase L1 gene in human airway epithelium of cigarette smokers. Cancer Res. 2006;66(22):10729–40. doi: 10.1158/0008-5472.CAN-06-2224 17108109

[53] Barrachina M, Castaño E, Dalfó E, Maes T, Buesa C, Ferrer I. Reduced ubiquitin C-terminal hydrolase-1 expression levels in dementia with Lewy bodies. Neurobiol Dis. 2006;22(2):265–73. doi: 10.1016/j.nbd.2005.11.005 16380264

[54] Todde V, Veenhuis M, van der Klei IJ. Autophagy: principles and significance in health and disease. Biochim Biophys Acta. 2009;1792(1):3–13. doi: 10.1016/j.bbadis.2008.10.016 19022377

[55] Cao Y, Klionsky DJ. Physiological functions of Atg6/Beclin 1: a unique autophagy-related protein. Cell Res. 2007;17(10):839–49. doi: 10.1038/cr.2007.78 17893711

[56] Levine B, Kroemer G. Autophagy in the pathogenesis of disease. Cell. 2008;132:27–42. 18191218 10.1016/j.cell.2007.12.018 PMC2696814

[57] Yang Z, Goronzy JJ, Weyand CM. Autophagy in autoimmune disease. J Mol Med (Berl). 2015;93(7):707–17. doi: 10.1007/s00109-015-1297-8 26054920 PMC4486076

[58] Zhao X, Zhang Q, Zheng R. The interplay between oxidative stress and autophagy in chronic obstructive pulmonary disease. Front Physiol. 2022;13:1004275. doi: 10.3389/fphys.2022.1004275 36225291 PMC9548529

[59] Li Y, Yin Y, Zhang T, Wang J, Guo Z, Li Y, et al. A comprehensive landscape analysis of autophagy in cancer development and drug resistance. Front Immunol. 2024;15:1412781. doi: 10.3389/fimmu.2024.1412781 39253092 PMC11381251

[60] Barmaki H, Nourazarian A, Khaki-Khatibi F. Proteostasis and neurodegeneration: a closer look at autophagy in Alzheimer’s disease. Front Aging Neurosci. 2023;15:1281338. doi: 10.3389/fnagi.2023.1281338 38020769 PMC10652403

[61] Hara T, Nakamura K, Matsui M, Yamamoto A, Nakahara Y, Suzuki-Migishima R, et al. Suppression of basal autophagy in neural cells causes neurodegenerative disease in mice. Nature. 2006;441(7095):885–9. doi: 10.1038/nature04724 16625204

[62] Komatsu M, Waguri S, Chiba T, Murata S, Iwata J, Tanida I, et al. Loss of autophagy in the central nervous system causes neurodegeneration in mice. Nature. 2006;441(7095):880–4. doi: 10.1038/nature04723 16625205

[63] Alirezaei M, Fox HS, Flynn CT, Moore CS, Hebb AL, Frausto RF, et al. Elevated ATG5 expression in autoimmune demyelination and multiple sclerosis. Autophagy. 2009;5:152–8. 19066443 10.4161/auto.5.2.7348 PMC2779564

[64] Rioux JD, Xavier RJ, Taylor KD, Silverberg MS, Goyette P, Huett A, et al. Genome-wide association study identifies new susceptibility loci for Crohn disease and implicates autophagy in disease pathogenesis. Nat Genet. 2007;39:596–604. 17435756 10.1038/ng2032 PMC2757939

[65] Saitoh T, Fujita N, Jang MH, Uematsu S, Yang B-G, Satoh T, et al. Loss of the autophagy protein Atg16L1 enhances endotoxin-induced IL-1beta production. Nature. 2008;456(7219):264–8. doi: 10.1038/nature07383 18849965

[66] Han J-W, Zheng H-F, Cui Y, Sun L-D, Ye D-Q, Hu Z, et al. Genome-wide association study in a Chinese Han population identifies nine new susceptibility loci for systemic lupus erythematosus. Nat Genet. 2009;41(11):1234–7. doi: 10.1038/ng.472 19838193

[67] International Consortium for Systemic Lupus Erythematosus Genetics (SLEGEN), Harley JB, Alarcón-Riquelme ME, Criswell LA, Jacob CO, Kimberly RP, et al. Genome-wide association scan in women with systemic lupus erythematosus identifies susceptibility variants in ITGAM, PXK, KIAA1542 and other loci. Nat Genet. 2008;40(2):204–10. doi: 10.1038/ng.81 18204446 PMC3712260

[68] Chen Z-H, Lam HC, Jin Y, Kim H-P, Cao J, Lee S-J, et al. Autophagy protein microtubule-associated protein 1 light chain-3B (LC3B) activates extrinsic apoptosis during cigarette smoke-induced emphysema. Proc Natl Acad Sci U S A. 2010;107(44):18880–5. doi: 10.1073/pnas.1005574107 20956295 PMC2973911

[69] Barnes PJ, Baker J, Donnelly LE. Autophagy in asthma and chronic obstructive pulmonary disease. Clin Sci (Lond). 2022;136:733–46. 35608088 10.1042/CS20210900 PMC9131388

[70] Ban G-Y, Pham DL, Trinh THK, Lee S-I, Suh D-H, Yang E-M, et al. Autophagy mechanisms in sputum and peripheral blood cells of patients with severe asthma: a new therapeutic target. Clin Exp Allergy. 2016;46(1):48–59. doi: 10.1111/cea.12585 26112695

[71] Jiang B, Zhou X, Yang T, Wang L, Feng L, Wang Z, et al. The role of autophagy in cardiovascular disease: Cross-interference of signaling pathways and underlying therapeutic targets. Front Cardiovasc Med. 2023;10:1088575. doi: 10.3389/fcvm.2023.1088575 37063954 PMC10090687

[72] Oka T, Hikoso S, Yamaguchi O, Taneike M, Takeda T, Tamai T, et al. Mitochondrial DNA that escapes from autophagy causes inflammation and heart failure. Nature. 2012;485(7397):251–5. doi: 10.1038/nature10992 22535248 PMC3378041

[73] Du J, Li Y, Zhao W. Autophagy and myocardial ischemia. Adv Exp Med Biol. 2020;1207:217–22. 32671750 10.1007/978-981-15-4272-5_15

[74] Poznyak AV, Nikiforov NG, Wu W-K, Kirichenko TV, Orekhov AN. Autophagy and Mitophagy as Essential Components of Atherosclerosis. Cells. 2021;10(2):443. doi: 10.3390/cells10020443 33669743 PMC7922388

[75] Bhattacharya D, Mukhopadhyay M, Bhattacharyya M, Karmakar P. Is autophagy associated with diabetes mellitus and its complications? A review. EXCLI J. 2018;17:709–20. doi: 10.17179/excli2018-1353 30190661 PMC6123605

[76] Kim J, Cheon H, Jeong YT, Quan W, Kim KH, Cho JM, et al. Amyloidogenic peptide oligomer accumulation in autophagy-deficient β cells induces diabetes. J Clin Invest. 2014;124(8):3311–24. doi: 10.1172/JCI69625 25036705 PMC4109549

[77] Shigihara N, et al. Human IAPP-induced pancreatic β cell toxicity and its regulation by autophagy. J Clin Invest. 2014;124:3634–44. 25036706 10.1172/JCI69866 PMC4109539

[78] Morita S, Sakagashira S, Shimajiri Y, Eberhardt NL, Kondo T, Kondo T, et al. Autophagy protects against human islet amyloid polypeptide-associated apoptosis. J Diabetes Investig. 2011;2(1):48–55. doi: 10.1111/j.2040-1124.2010.00065.x 24843461 PMC4008015

[79] Hanna J, Guerra-Moreno A, Ang J, Micoogullari Y. Protein Degradation and the Pathologic Basis of Disease. The American Journal of Pathology. 2019;189:94–103. 30312581 10.1016/j.ajpath.2018.09.004 PMC6315326

[80] Dantuma NP, Bott LC. The ubiquitin-proteasome system in neurodegenerative diseases: precipitating factor, yet part of the solution. Front Mol Neurosci. 2014;7:70. doi: 10.3389/fnmol.2014.00070 25132814 PMC4117186

[81] Zheng Q, Huang T, Zhang L, Zhou Y, Luo H, Xu H, et al. Dysregulation of Ubiquitin-Proteasome System in Neurodegenerative Diseases. Front Aging Neurosci. 2016;8:303. doi: 10.3389/fnagi.2016.00303 28018215 PMC5156861

[82] Park J, Cho J, Song EJ. Ubiquitin-proteasome system (UPS) as a target for anticancer treatment. Arch Pharm Res. 2020;43(11):1144–61. doi: 10.1007/s12272-020-01281-8 33165832 PMC7651821

[83] Suh KS, Tanaka T, Sarojini S, Nightingale G, Gharbaran R, Pecora A, et al. The role of the ubiquitin proteasome system in lymphoma. Crit Rev Oncol Hematol. 2013;87:306–22. 23541070 10.1016/j.critrevonc.2013.02.005

[84] Zhang X, Linder S, Bazzaro M. Drug development targeting the ubiquitin-proteasome system (UPS) for the treatment of human cancers. Cancers. 2020;12. 10.3390/cancers12040902 PMC7226376 32272746

[85] Zhang Y, Du L, Wang C, Jiang Z, Duan Q, Li Y, et al. Neddylation is a novel therapeutic target for lupus by regulating double negative T cell homeostasis. Signal Transduct Target Ther. 2024;9(1):18. doi: 10.1038/s41392-023-01709-9 38221551 PMC10788348

[86] Verbrugge SE, Scheper RJ, Lems WF, de Gruijl TD, Jansen G. Proteasome inhibitors as experimental therapeutics of autoimmune diseases. Arthritis Res Ther. 2015;17(1):17. doi: 10.1186/s13075-015-0529-1 25889583 PMC4308859

[87] Powell SR, Herrmann J, Lerman A, Patterson C, Wang X. The ubiquitin-proteasome system and cardiovascular disease. Prog Mol Biol Transl Sci. 2012;109:295–346. 22727426 10.1016/B978-0-12-397863-9.00009-2 PMC3743449

[88] Wing SS. The UPS in diabetes and obesity. BMC Biochem. 2008;9 Suppl 1(Suppl 1):S6. doi: 10.1186/1471-2091-9-S1-S6 19007436 PMC2582800

[89] Liu Z, Wang P, Zhao Y, Po Lai K, Li R. Biomedical importance of the ubiquitin-proteasome system in diabetes and metabolic transdifferentiation of pancreatic duct epithelial cells into β-cells. Gene. 2023;858:147191. doi: 10.1016/j.gene.2023.147191 36632913

[90] Gouveia M, Teixeira M, Schmidt C, Lopes M, Trindade D, Magalhães S, et al. Impaired Extracellular Proteostasis in Patients with Heart Failure. Arch Med Res. 2023;54(3):211–22. doi: 10.1016/j.arcmed.2023.02.001 36797157

[91] Radisky ES. Extracellular proteolysis in cancer: Proteases, substrates, and mechanisms in tumor progression and metastasis. J Biol Chem. 2024;300(6):107347. doi: 10.1016/j.jbc.2024.107347 38718867 PMC11170211

[92] Piñero J, Bravo À, Queralt-Rosinach N, Gutiérrez-Sacristán A, Deu-Pons J, Centeno E, et al. DisGeNET: a comprehensive platform integrating information on human disease-associated genes and variants. Nucleic Acids Res. 2017;45(D1):D833–9. doi: 10.1093/nar/gkw943 27924018 PMC5210640

[93] Liang XH, Jackson S, Seaman M, Brown K, Kempkes B, Hibshoosh H, et al. Induction of autophagy and inhibition of tumorigenesis by beclin 1. Nature. 1999;402(6762):672–6. doi: 10.1038/45257 10604474

[94] Chen N, Debnath J. Autophagy and tumorigenesis. FEBS Lett. 2010;584(7):1427–35. doi: 10.1016/j.febslet.2009.12.034 20035753 PMC2843775

[95] Liang C, Jung JU. Autophagy genes as tumor suppressors. Curr Opin Cell Biol. 2010;22:226–33. 19945837 10.1016/j.ceb.2009.11.003 PMC2854193

[96] Degenhardt K, Mathew R, Beaudoin B, Bray K, Anderson D, Chen G, et al. Autophagy promotes tumor cell survival and restricts necrosis, inflammation, and tumorigenesis. Cancer Cell. 2006;10(1):51–64. doi: 10.1016/j.ccr.2006.06.001 16843265 PMC2857533

[97] Mathew R, et al. Autophagy suppresses tumorigenesis through elimination of p62. Cell. 2009;137:1062–75. 19524509 10.1016/j.cell.2009.03.048 PMC2802318

[98] Fung C, Lock R, Gao S, Salas E, Debnath J. Induction of autophagy during extracellular matrix detachment promotes cell survival. Mol Biol Cell. 2008;19(3):797–806. doi: 10.1091/mbc.e07-10-1092 18094039 PMC2262959

[99] Mowers EE, Sharifi MN, Macleod KF. Autophagy in cancer metastasis. Oncogene. 2017;36(12):1619–30. doi: 10.1038/onc.2016.333 27593926 PMC5337449

[100] Marsh T, Tolani B, Debnath J. The pleiotropic functions of autophagy in metastasis. J Cell Sci. 2021;134(2):jcs247056. doi: 10.1242/jcs.247056 33483365 PMC7847272

[101] Dower CM, Wills CA, Frisch SM, Wang H-G. Mechanisms and context underlying the role of autophagy in cancer metastasis. Autophagy. 2018;14(7):1110–28. doi: 10.1080/15548627.2018.1450020 29863947 PMC6103720

[102] Polara R, van Rinsum D, Robinson N. Autophagy in cancer metastasis. In: Shravage BV, Turksen K. Autophagy in stem cell maintenance and differentiation. Cham: Springer International Publishing. 2023;259–85.

[103] Zhou X, Xu R, Wu Y, Zhou L, Xiang T. The role of proteasomes in tumorigenesis. Genes Dis. 2023;11(4):101070. doi: 10.1016/j.gendis.2023.06.037 38523673 PMC10958230

[104] Deng L, Meng T, Chen L, Wei W, Wang P. The role of ubiquitination in tumorigenesis and targeted drug discovery. Signal Transduct Target Ther. 2020;5(1):11. doi: 10.1038/s41392-020-0107-0 32296023 PMC7048745

[105] Hoeller D, Dikic I. Targeting the ubiquitin system in cancer therapy. Nature. 2009;458(7237):438–44. doi: 10.1038/nature07960 19325623

[106] Demo SD, Kirk CJ, Aujay MA, Buchholz TJ, Dajee M, Ho MN, et al. Antitumor activity of PR-171, a novel irreversible inhibitor of the proteasome. Cancer Res. 2007;67(13):6383–91. doi: 10.1158/0008-5472.CAN-06-4086 17616698

[107] D’Arcy P, Brnjic S, Olofsson MH, Fryknäs M, Lindsten K, De Cesare M, et al. Inhibition of proteasome deubiquitinating activity as a new cancer therapy. Nat Med. 2011;17(12):1636–40. doi: 10.1038/nm.2536 22057347

[108] Wang X, Mazurkiewicz M, Hillert E-K, Olofsson MH, Pierrou S, Hillertz P, et al. The proteasome deubiquitinase inhibitor VLX1570 shows selectivity for ubiquitin-specific protease-14 and induces apoptosis of multiple myeloma cells. Sci Rep. 2016;6:26979. doi: 10.1038/srep26979 27264969 PMC4893612

[109] Soucy TA, Smith PG, Milhollen MA, Berger AJ, Gavin JM, Adhikari S, et al. An inhibitor of NEDD8-activating enzyme as a new approach to treat cancer. Nature. 2009;458(7239):732–6. doi: 10.1038/nature07884 19360080

[110] Vassilev LT, Vu BT, Graves B, Carvajal D, Podlaski F, Filipovic Z, et al. In vivo activation of the p53 pathway by small-molecule antagonists of MDM2. Science. 2004;303(5659):844–8. doi: 10.1126/science.1092472 14704432

[111] Ling S-C, Polymenidou M, Cleveland DW. Converging mechanisms in ALS and FTD: disrupted RNA and protein homeostasis. Neuron. 2013;79(3):416–38. doi: 10.1016/j.neuron.2013.07.033 23931993 PMC4411085

[112] Dickens JA, Malzer E, Chambers JE, Marciniak SJ. Pulmonary endoplasmic reticulum stress-scars, smoke, and suffocation. FEBS J. 2019;286(2):322–41. doi: 10.1111/febs.14381 29323786

[113] Tran I, Ji C, Ni I, Min T, Tang D, Vij N. Role of Cigarette Smoke-Induced Aggresome Formation in Chronic Obstructive Pulmonary Disease-Emphysema Pathogenesis. Am J Respir Cell Mol Biol. 2015;53(2):159–73. doi: 10.1165/rcmb.2014-0107OC 25490051 PMC5455694

[114] Guerra J, Matta L, Bartelt A. Cardiac proteostasis in obesity and cardiovascular disease. Herz. 2024;49(2):118–23. doi: 10.1007/s00059-024-05233-6 38329532 PMC10917825

[115] Cavadas C, Aveleira CA, Souza GFP, Velloso LA. The pathophysiology of defective proteostasis in the hypothalamus - from obesity to ageing. Nat Rev Endocrinol. 2016;12(12):723–33. doi: 10.1038/nrendo.2016.107 27388987

[116] Kamya P, Ozerov IV, Pun FW, Tretina K, Fokina T, Chen S, et al. PandaOmics: An AI-Driven Platform for Therapeutic Target and Biomarker Discovery. J Chem Inf Model. 2024;64(10):3961–9. doi: 10.1021/acs.jcim.3c01619 38404138 PMC11134400

[117] Ritchie ME, Phipson B, Wu D, Hu Y, Law CW, Shi W, et al. limma powers differential expression analyses for RNA-sequencing and microarray studies. Nucleic Acids Res. 2015;43(7):e47. doi: 10.1093/nar/gkv007 25605792 PMC4402510

