# Supplementary figures and images for: Proteostasis signatures in human diseases
Source: PLoS Comput Biol. 2025 Jun 17;21(6):e1013155. doi: 10.1371/journal.pcbi.1013155 (PMC12173376; doi:10.1371/journal.pcbi.1013155)

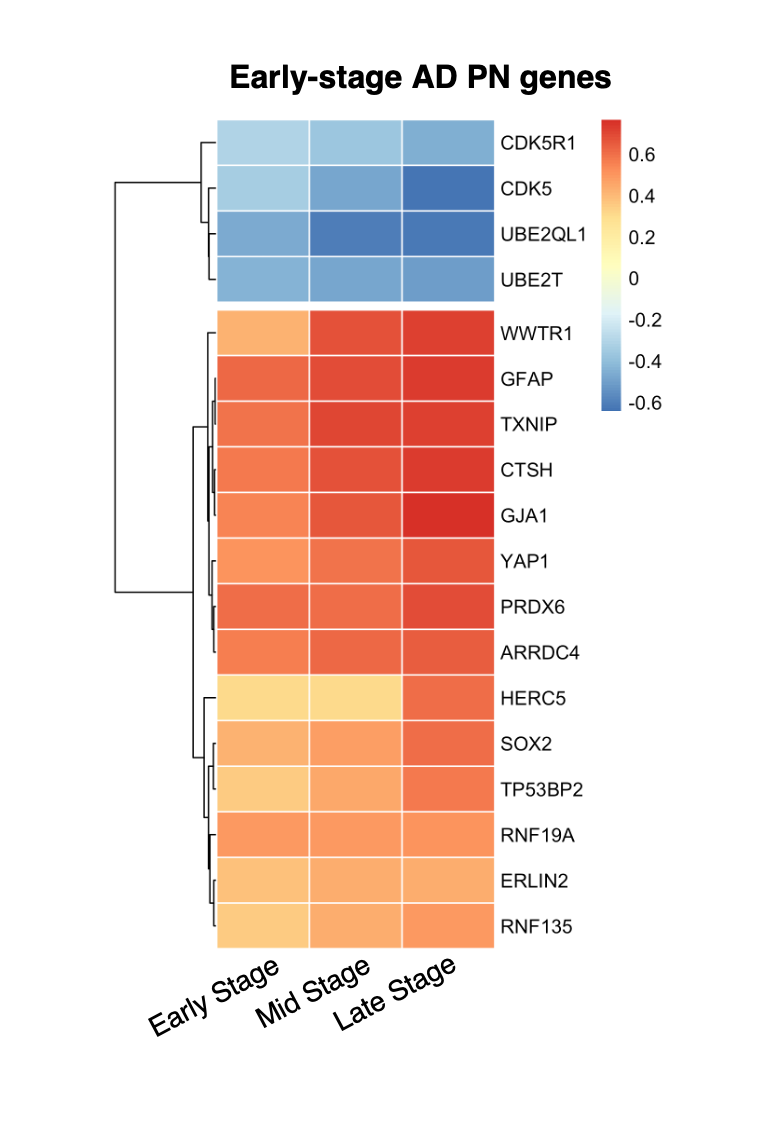

Supplement: S1 Fig — Red indicates upregulation of a PN gene compared to control. Blue indicates downregulation of a PN gene compared to control. Intensity depicts extent of log2foldchange. (PNG) [file pcbi.1013155.s001.png]
